# Pregnancy in Disability: Community Perceptions and Personal Experiences in a Rural Setting in Ghana

**DOI:** 10.1155/2018/8096839

**Published:** 2018-12-16

**Authors:** Bridget Dela Akasreku, Helen Habib, Augustine Ankomah

**Affiliations:** ^1^Department of Population, Family and Reproductive Health, School of Public Health, College of Health Sciences, University of Ghana, Accra, Ghana; ^2^Department of Epidemiology and Disease Control, School of Public Health, College of Health Sciences, University of Ghana, Accra, Ghana

## Abstract

**Background:**

Persons with disabilities (PWDs) generally face strong discrimination and exclusion, especially with regard to their sexual and reproductive health. There is a prevailing social myth in Ghana that women with disabilities are asexual and cannot experience a positive motherhood experience. Although the World Health Organization recommends that research is conducted in this regard, community attitudes towards pregnant women living with disabilities remain largely unexplored. The purpose of this study was to explore community attitudes to pregnancy among women living with disabilities.

**Methodology:**

The study design was a mixed method cross-sectional study involving quantitative face-to-face individual interviews with 400 randomly selected community members (both males and females) in three communities in the Adaklu District of Volta Region in Ghana. In addition, in-depth interviews were held with five female PWDs. Simple and multiple logistic regression analysis were used to examine the factors associated with perceptions towards pregnant PWDs.

**Results:**

Majority of respondents agreed that pregnant women with disabilities should be kept in special institutions until delivery to prevent transmission of their disability to fetuses of pregnant women without disabilities. People also believed that pregnant PWDs are incapable of a safe motherhood experience. Among the strongly influencing factors for negative attitudes towards pregnancy of PWDs were educational status (p<0.001) and perceptions that the disability is caused by accidents (p<0.001) or spiritual issues (p<0.01). Regarding the relationship between perceived cause of disability and the resultant attitudes, respondents were three times more likely to have negative attitude and perceptions towards pregnant women with disabilities if their causes of disabilities were perceived to be spiritual compared to the cause being medical.

**Conclusion:**

Our findings indicate that there are generally negative societal attitudes towards pregnant PWDs. The evidence suggests that a degree of prejudice and misconceptions exists towards the pregnancy of women living with disabilities. Generally, there is a public perception that women living with disabilities cannot have a safe motherhood experience and are capable of transferring their disability to an unborn child of another pregnant woman.

## 1. Background

Globally, approximately 785 million (15.6%) persons 15 years and older live with a disability [1]. The United Nations Development Programme (UNDP) [2] estimates that 80% of these people live in developing countries [3] with 65% to 70% being women [4]. Although persons with disabilities (PWDs) face discrimination and social, cultural, and economic exclusion worldwide, women with disabilities experience unique and additional disadvantages because of intersectional discrimination of gender and disability [5, 6]. Resultantly, they are more likely to experience social exclusion compared with their male counterparts [6]. This exclusion compromises a number of life outcomes for female PWDs including sexual and reproductive health (SRH) [6, 7].

The United Nations Convention on the Rights of Persons with Disabilities (CRPD) has specific provisions that recognize the reproductive rights of PWDs (Art. 23) [2]. In developing countries such as Ghana, efforts to uphold these rights are hampered by negative public perceptions about the reproductive lives of female PWDs [8]. Evidence suggests that factors that undermine the reproductive health of women with disabilities are multifaceted [5, 9, 10]. These are evident at the family and community levels where sociocultural norms and beliefs deny female PWDs the right to exercise their reproductive rights and the national level where maternal health interventions hardly address the concerns of female PWDs [5, 9, 10]. Even where services are available, it is necessary to understand how service providers and the general society can support the SRH of women with disabilities [5, 9, 10]. It is evident that most Ghanaian infrastructure is unfriendly towards persons with disabilities [10]. Most public facilities and services including healthcare facilities are structurally inaccessible to PWDs [10]. The Persons with Disability Act, 2006 (Act 715), in Ghana defines a person living with disability as,* “a person with a physical, mental or sensory impairment including a visual, hearing, or speech functional disability, which gives rise to physical, cultural, or social barriers that substantially limits one or more of the major life activities of that individual” *[11]. The World Health Organization (WHO) estimates disability rate in Ghana to be between 6 and 10 percent which represents approximately 1.5–2.2 million people of the Ghanaian population [12] although data from Ghana reports figures only half as high [13]. Additionally, population-targeted interventions, especially in healthcare, are still unpopular among PWDs [12]. Furthermore, there is low societal awareness about the laws that protect the rights of PWDs, even among PWDs themselves and their close relatives and other care givers. Consequently, PWDs and their relatives and caregivers usually do not realize when they are denied their basic rights.

The Ghana Statistical Service estimates that more than half of Ghana's population who live with disabilities in Ghana are women [13]; many of whom are disproportionately affected by poverty [1]. According to 2010 Ghana Population and Housing Census, 737,743 of the Ghanaian population (3.0% of the total population) are PWDs [13]. In the Volta region (one of Ghana's ten administrative regions where the study took place), 4.3% of the population are PWDs (the highest proportion in the country) [13] and there are several disability help groups [14]. Despite Ghana's ratification of the convention on the rights of PWDs (CRPD) in 2006 and the existence of legal frameworks that uphold the reproductive rights of women with disabilities, Ghanaian female PWDs continue to experience high rates of SRH rights violations [11]. Existing research shows that female PWDs equally desire sex, pregnancy, and a positive motherhood experience as women without disabilities. However, in Ghana, several factors undermine the sexual and reproductive health of women with disabilities. One identified factor is inaccurate and negative stereotypes, including perceptions that women with disabilities cannot have a positive motherhood experience. These perceptions have resulted in generally negative public attitudes towards pregnant PWDs [10, 15–18]. Also, many are faced with social barriers during pregnancy given the various societal prejudices, myths, and misconceptions [10]. These societal issues are further entrenched by cultural and religious beliefs that perceive a disability as a curse or a punishment for a bad deed by a PWDs or their close relative or ancestors [16, 19]. Ghanaian communities generally signify pregnancy and childbirth as blessings, but the birth of a disabled child is usually ascribed to a punishment and curse of an angry god [16, 19]. These beliefs generally lead to pregnant women being subjected to several taboos and prohibitions including the prohibition of nutritious food such as eggs, with the belief of warding off the birth of a child with a disability [16]. The birth of a child with a disability is mostly attributed to violation of traditional norms and taboos [16]. Pregnant women without disabilities are therefore strongly advised by friends and family to avoid contact with pregnant women with disabilities to prevent the transfer of a disability to their unborn child [16]. Following these spiritual and cultural beliefs, women with disabilities in Ghana face strong social barriers and discrimination during pregnancy [16, 19]. In certain communities, children with disabilities are left at shrines to correct these “bad omens” [16]. If a child dies in the process, it is believed the child was not meant for this world [19]

In addition to societal barriers, women with disabilities face barriers at healthcare institutions [6, 10, 20]. Firstly, studies suggest that maternal and reproductive health care interventions are least targeted at women with disabilities [10, 21]. For most women or girls living with disabilities, knowledge on SRH and rights is very poor, coupled with limited access to sexuality information [6, 10, 20]. It was revealed in a Ghanaian study that healthcare providers' insensitivity and lack of knowledge on the maternal health needs of pregnant women with disabilities was a major barrier faced by women with disabilities in accessing SRH services in Ghana [10]. For instance, PWDs highlighted healthcare providers' lack of knowledge of basic sign language, creating a significant communication gap [10]. Pregnant women with disabilities have reported challenges with doctors and nurses' inability to understand explanations about their maternal health history which has often times resulted in wrong prescriptions and medical treatment [21].

Additionally, the format in which pregnancy-related information is presented coupled with the unavailability of assistive devices like braille, audio, and sign interpreters restricts access to SRH information [20]. Ganle et al. in their study among women with disabilities [10] reported that information from healthcare providers is less accessible to women with disabilities [10]. For instance, individuals with visual impairments find healthcare information that are embedded in pictures and on flip charts recondite; as this information is not presented in braille. Tun et al. in their study similarly reported that people with disabilities have only a limited amount of information in accessible formats about SRH services including HIV counseling and testing [22]. Group messages and maternal health education given during outpatient services and antenatal care services, likewise, have been reported to be inaccessible for individuals with hearing impairments [10]. These challenges faced at health institutions often discourage pregnant women with disabilities from using facility-based reproductive health services [23], leading to many poor pregnancy outcomes [10].

In 2009, WHO provided guidelines promoting the SRH rights of PWDs to help minimize negative societal perceptions and attitudes towards PWDs. One of the components of this framework is to promote research at the local, national and international levels that seek to investigate both social and individual factors that explain these negative attitudes [1]. Community level studies have assessed levels of perceptions in countries including Australia [24]. Over the years, research on the reproductive health of PWDs in Ghana has focused on the challenges faced by pregnant women with disabilities while accessing maternal health services. Community attitudes and perceptions towards pregnant PWDs remain largely unexplored. For communities to acknowledge the reproductive rights of women with disabilities, it is necessary to understand the social barriers female PWDs face and the negative perceptions and stereotypes among various communities which stigmatize women with disabilities. This study, therefore, explores attitudes and perceptions towards pregnant PWDs to inform policy development and implementation towards the promotion of the SRH needs and rights of women with disabilities. This may contribute to better understanding the determinants of maternal health disparities and help advance the goal of bridging these disparities especially among women with disabilities.

## 2. Methods

The study design was a mixed method cross-sectional study involving quantitative face-to-face individual interviews with community members without disabilities and qualitative in-depth interviews with female PWDs. The study location was the Adaklu District, in Volta Region of Ghana. The community is predominantly rural with the majority of the population engaged in agricultural, forestry, and fishery services. Data collection took place in June 2017. The interviews were conducted in person firstly because of the sensitive nature of the topic of interest and also because of unstable phone and Internet access in the community which would interfere with Internet or phone based surveys. Face-to-face surveys also allowed for people without reading and writing literacy to be interviewed, which would have been a challenge in self-administered or mailed surveys. Additionally, the personal nature of the interviewers allowed for nonverbal cues to be observed and probed for richer qualitative responses.

### 2.1. Study Population and Sample Size

The study populations were community residents (males and females) between ages 18 years and 65 years. Participants for the in-depth interview were five (5) consenting female PWDs who have experienced pregnancy (and who live with at least one physical, sight, or hearing impairment) from within the district, within the specified age range who could provide in-depth information on their experiences concerning pregnancy of PWDs. We based our sample size calculation on a study in Ghana on the proportion of respondents that believe people with disabilities receive unfair treatment, reported to be 60% [16]. The sample size was computed using Cochran's formula (1965) as follows:

(1) $$\begin{array}{c} N=\frac{Z^{2}*\left(p\right)\left(q\right)}{d^{2}} \end{array}$$

N= required sample size,
Z=1.96 (at 95% confidence interval),
P=Prevalence of unfair treatment, d=Margin of error,
  (2) $$\begin{array}{c} N=\frac{1.96^{2}*\left(0.6\right)\left(1-0.6\right)}{0.05^{2}} \end{array}$$

With the parameters, the sample size when calculated was 368. The total sample size was estimated to be 400 assuming a 10% nonresponse rate.

### 2.2. Quantitative Data Collection and Analysis

Multistage random sampling was employed in selecting participants for the quantitative component of the study. The 2010 Ghana Population and Housing Census estimated the total population of study district to be 36,391 with twenty communities (all rural). These communities were grouped into large and small communities based on their population sizes.

A population size below 1,200 inhabitants was classified as “small” whereas 1,200 or more was classified as “large”. A total of three communities were randomly selected for the study: one large community and two small communities. With the help of three research assistants and a town council member, houses were listed from each community using sequential system of numbering and counting. A systematic sampling with a random start was used to select houses for interviews.

Males and females were proportionately selected. Questionnaires were pretested at a nonstudy community within the district followed by minor changes to the questionnaire. Interviews were administered by three trained research assistants under the supervision of the principal investigator. Interviews were conducted in the evenings, when most community members were back from work. Completed forms were reviewed daily and on-the-spot feedback provided with follow-up or call-back checks, where necessary.

The questionnaire consisted of three parts: (1) sociodemographic characteristics (including age, educational status, sex, and occupation) and other factors such as cultural beliefs and taboos and respondent's awareness of disability rights; (2) a range of questions to identify respondents' perceptions towards pregnant women living with disabilities; and (3) a list of statements to measure respondents' acceptance level of pregnancy among women with disabilities. Data were coded and entered into Excel and subsequently exported into Stata 14 for analysis.

To measure respondents' attitudes to pregnancy in disability (dependent variable), a composite measure of attitude for each respondent was generated. In all, a total of 19 items were scored. The theoretical scores ranged from 1 to 19 but the actual scores ranged from 9 to 19. Scores were dichotomized into positive acceptance and negative acceptance using a median split of 15, indicating <15 as negative attitudes (low acceptance level) and ≥15 as positive attitude (high acceptance level). Details of items are presented in Table 4. Exposure to a woman with disability was measured by asking whether respondents live in the same house with women with disability or has a female relative or friend with disability or works with a woman with disability.

Frequency distribution tables, means, percentages, and cross tabulations were used to investigate and describe variables. Statistically, differences in demographic characteristics about attitudes and perceptions towards pregnant women with disabilities (dependent variable) were analysed in a bivariate analysis. Pearson chi-square statistics were used to determine explanatory variables that were statistically significant. A multiple logistic regression analysis was conducted. A p-value < 0.05 was used to denote statistical significance.

### 2.3. Qualitative Component

A purposive sampling technique was used for the qualitative component. Respondents were identified through contacts with the Voice of People with Disability, Ghana (VOICE GHANA), a registered Ghanaian nongovernmental organization. Five (5) women living with disabilities were selected according to age, disability type and community of residence. Interviews were conducted in the local Ewe language. An interview guide focused on various community perceptions, acceptance, and attitudes identified or experienced by women living with disabilities with regard to their pregnancy was used. Interviews were recorded with a tape recorder with a note-taker present. Interviews lasted around an hour. Transcribed texts were validated by the first author, a native speaker of the local language. Translated data were thereafter imported into Nvivo 10. Imported text was organized and coded and patterns and interconnections were built. The data was then organized thematically with evolving quotes categorized under generated themes and subthemes.

## 3. Ethical Consideration

Approval was granted from the Ghana Health Service Ethical Review Committee. Written consent was obtained from participants after explaining the purpose of the study as well as their participatory rights, risks, and benefits. Interviews were conducted individually. Completed questionnaires were coded with unique ID numbers, signed by research assistants and safely transferred to the first author for data entry. Data transferred onto a computer was secured with a password. The in-depth interviews (IDIs) were digitally recorded with the permission of the participant. All recordings were safely kept under lock and key is only accessible to the research team.

## 4. Results

### 4.1. Background and Sociodemographic Characteristics

Table 1 represents sociodemographic characteristics of the 400 respondents surveyed. Slightly more than half (55.2%) of the respondents were females. The mean age of respondents was 30 ± 8 years. The largest percentage of respondents was cohabiting (42.6%) with 26.7% being married. Majority (86.1%) had some formal education with three-quarters (74.5%) being employed. In terms of tribe, respondents were predominantly Ewes (97.2%).

### 4.2. Characteristics of Respondents Living with Disabilities

Three (3) of the IDI respondents were physically impaired while two (2) had visual and hearing impairments. Ages ranged from 18 to 65 years. None of these respondents were married at the time of the study although two (2) had been previously married. Four (4) respondents had received some level of formal education and were employed. Almost all the respondents (4) lived with their family members and had never resided outside their family homes since childhood.

### 4.3. Awareness of the Existence of Disability Laws or Rights and Exposure to PWDs

Respondents were asked whether they knew of the existence of legislation on PWDs. Only 3.5% reported having knowledge on the existence of such law. Having personal contacts with PWDs has been reported to inform perceptions and attitudes towards PWDs. Qiaolan et al. [25] have reported how prolonged and frequent contacts with PWDs have resulted in negative attitudes towards PWDs. In view of this, our study asked respondents to mention whether they have any personal contacts with women with disabilities. As shown in Table 2, 12.8% of respondents mentioned that they have ever had close personal contact with female PWDs (e.g., as family members, neighbours, and members of a community group).

### 4.4. Respondents Perceived Cause of Disability

Respondents were asked what they thought causes disability. Multiple reasons were given (Table 2). The top three mentioned causes of disabilities were spiritual (88.8%) followed by accidents (85.8%) and medical condition (46.0%). This was in agreement with findings from IDIs; one vital theme being the cultural and traditional belief that disability had a spiritual origin. For example, a PWD said:

*“In this community, disability is perceived to be transferred spiritually through birth. It is believed that a pregnant PWD may transfer her disability to the unborn child. I think that is why the members of this community discourage pregnancy among PWDs”* (PWD5).

### 4.5. Perception on Pregnancy

Respondents were asked how they felt whenever they met pregnant women with disabilities. Responses are shown in Table 3. Most of the respondents (69.9%) said that they were felt uncomfortable or awkward whenever they came across pregnant PWDs although 38.8% said that they were not afraid. Only 9.3% percent indicated that they never felt uncomfortable upon meeting a pregnant PWD. Majority of the respondents (60.3%) felt sorry for pregnant women living with disabilities.

### 4.6. Overall Accepting Attitudes towards Pregnant Women with Disabilities

To gauge levels of accepting attitudes towards pregnancy in disability, respondents were asked to indicate whether they agreed or disagreed with certain statements about pregnant PWDs. Nearly all respondents (99.5%) agreed that women with disabilities can have children. Nonetheless, nearly three-quarters of respondents (72.3%) were of the opinion that pregnant PWDs are incapable of having a positive labor and childbirth experience. Even though majority of respondents (86.5 percent) initially thought that female PWDs should not be kept apart from the rest of society, 95.7 percent of these respondents thought that they should be kept in special societal institutions during pregnancy till child birth. As a corollary, more than half of the respondents (56.3%) thought that pregnant women without disabilities should avoid close contact with pregnant PWDs to avoid the transmission of a disability to their unborn child. This was confirmed by women with disabilities themselves:

*Even during antenatal care, pregnant women without disabilities did not want to touch me, because they thought they might give birth to something of my sort (PWD3)*.

Nearly three-quarters (72.3%) of community respondents agreed that pregnant PWDs are incapable of going through child birth with little or no related complications, which was confirmed by PWDs themselves.

*“My boyfriend's relatives were not prepared to handle my pregnancy. They expressed their displeasure about my pregnancy. One of them said to me: ‘Why can't you have pity on yourself? Don't you know pregnancy is meant for abled bodies?”* (PWD3).

Data from the qualitative interviews show that some health care workers hold negative attitude and misconceptions about disability and pregnancy. Women with disabilities reported to have encountered or seen a fellow woman with a disability having had unpleasant encounters with health care providers, where providers expressed concerns about the incapability of pregnant PWDs to have a positive or safe motherhood experience. The following quotes typified encounters with service providers.

*“The midwife was not willing to handle my pregnancy. As soon as she saw me, she tagged my pregnancy as high risk without critical physical examination. She then referred me to a different facility which I never went. Eventually, I delivered my baby at home” (PWD3)*.

In a related way, there was the perception that female PWDs are not best fit for marriage even if they could be sexual partners; a large majority (94.5%) of survey respondents agreed with the statement that a woman with a disability can marry a man without a disability. Nonetheless, IDIs with women with disabilities revealed the contrary. Four out of five respondents asserted that it is difficult for women living with disabilities to marry men without disabilities. A respondent stated “*My fiancée broke up his relationship with me and chose to marry another woman rather than me. He knew his family may not support his decision to marry a woman in a wheelchair”; as women in wheel chairs in our community are perceived as incapable to giving birth” (PWD3) *

Another respondent had this to say:* “Men often come to me for only sex. None of them wants to get married to me. They are afraid of being laughed at in society as having married a woman with a disability who can't even give birth or take care of a child.”* (PWD1).

We went further to ask respondents' their perceptions about the sexuality and marriage of female PWDs as these has been reported in previous studies [10, 21] to be one of the major facilitators to forming negative perceptions and attitudes about women with disabilities and their pregnancies. This section presents respondents' perceptions about the sexuality of women living with disabilities and their pregnancies. Respondents were asked to indicate whether they “agreed or disagreed” with certain statements on the sexuality of women living with disabilities. The findings are presented in Table 4. Majority of the respondents (60.8%) agreed to the statement that women with disabilities are asexual. Similarly, a large proportion of respondents (70.5%) agreed to the statement that women living with disabilities cannot have sexual feelings or desires. In addition, less than half (43.8%) would agree to a relative to have sex with a woman with disability. These views were further supported by our in-depth interviews with women with disabilities. A recurring theme that emerged in this regard was the public perception that women with disabilities are asexual. They are not expected to have sex let alone become pregnant. The following quotes exemplified the public perception that women living with disabilities are sexually unattractive and less worthy to experience any form sexual desire. This was confirmed by women with disabilities themselves:

*I have been an involuntary celibate for several years. There are times I wished l had a sexual partner but because I'm always in a wheelchair, I don't think I fit as an ideal lady who can sexually attract a man (PWD2). *Another PWD had this to say:* One time I heard some ladies gossiping about me. One said “she has a pretty face. She would have been so sexy but for her disability, she isn't” (PWD4)*.

While a great majority (94.5%) of respondents felt that a female PWD can marry a man without a disability and 94.8% disagreed that a woman with a disability can only have sexual relations with a man with a disability, more than half of that figure (56.3%) disagreed that they were comfortable with their close friend or relative having a sexual relationship with a female PWD and even less (39.2%) agreed they would be comfortable having a PWD as a sister-in-law

*“One day my sexual partner took me to his house to introduce me to his family. As soon as his mother saw us, she gave an unreceptive facial expression and said to her son (my fiancée) “so upon all the women in this world, is this type of woman you would like to marry? What help can she offer you? I felt very embarrassed and never stepped my feet in that house again. Our relationship was kept a secret from family and friends since then”* (PWD3).

### 4.7. Relationship between Sociodemographic and Other Factors and Attitudes towards Pregnant Women with Disabilities

At the bivariate level, the study sought to explore the relationships between selected sociodemographic characteristics and attitude towards pregnant PWDs (see Table 5). To measure respondents' attitude, a composite measure of attitude was generated and dichotomized. Overall, a majority of respondents (66.3%) had negative accepting attitudes towards pregnant women with disabilities. There was a statistically significant association (p<0.001) between education and attitudes towards pregnancy with disability. The lower the education, the higher the level of nonaccepting (negative) attitudes. However, there was no significant relation between sex (*p=*0.074) or age (p=0.115) and attitudes and perceptions towards pregnant PWDs A strong predictor (p=0.022) for negative attitude towards PWDs was exposure; among people who had exposure to PWDs, 80.4% had a negative attitude to PWDs while 64% of people who had no exposure to PWDs scored negative attitudes. However, knowledge of laws about PWDs was a strong predictor for positive attitudes. More than half (57.1%) of people who knew about disability laws had a positive attitude towards PWDs while only a third (32.7%) of those who had no knowledge on disability laws reported positive attitudes (p=0.057).

Table 5 also presents a bivariate analysis of other factors and negative attitudes and perceptions of respondents towards pregnant women with disabilities. Respondents who perceived disability as a result of spiritual issues and accidents were also more likely to have negative attitudes to PWDs (accidents* p*<0.001 and spiritual causes* p*<0.01). Similarly, respondents who had been exposed or have had close contact with PWDs were more likely to have accepting attitudes towards pregnant PWDs (*p*<0.05). It is important to note that none of the key personal characteristics, sex, age, marital status, and employment status, was statistically associated with attitudes towards pregnancy in disability.

### 4.8. Predictors of Negative Attitude and Perceptions towards Pregnant Women with Disabilities

The multiple logistic regression models examined the predictors of attitude and perceptions of respondents.  Table 6 presents results from the logistic regression model with crude (unadjusted) and adjusted odd ratios. We included in the model only variables which were significant at p<0.1 at the bivariate level. The results show that the most significant variables that influenced respondents' attitude and perceptions towards pregnant PWDs were beliefs that disabilities were caused by accidents (AOR=5.68, 95% CI=2.22-14.55) and spiritual conditions (AOR=3.16, 95%* CI*=1.37-7.26) as well as respondents' level of awareness of disability laws and rights (AOR=4.21, 95%* CI*=1.18-15.03). Respondents were less likely to exhibit negative attitude towards pregnant PWDs whose disabilities were perceived to have been caused by medical conditions (A*OR*=0.34, 95% CI*= *0.20-0.56) but more than five times likely to exhibit negative attitude and perceptions towards accident-caused disability (AOR=5.68, 95% CI=2.22-14.55). Similarly, respondents were about 3 times more likely to have negative attitudes towards perceived spiritually caused disability (AOR=3.16, 95% CI=1.37-7.26). Respondents who were not aware of laws and rights of PWDs were more than 4 times likely to exhibit negative attitude and perceptions towards pregnant PWDs as opposed to those who were aware (*OR*=4.21, 95% CI=1.18-15.03). There was no evidence that sex was a statistically significant indicator in explaining accepting attitudes towards pregnancy in disability. Respondents who had attained at least secondary level of education were twice as likely to have negative attitude and perceptions towards pregnant women with disabilities as compared to those with at most primary level of education.

## 5. Discussion

We carried out this study to explore community attitude to pregnancy among women living with disabilities. More than half of our survey respondents believed that pregnant women without disabilities should avoid direct contact with pregnant PWDs because disability is spiritually transmissible to the fetus of a pregnant woman by direct contact with a PWD. An almost universal view was that society should keep pregnant PWDs in special institutions till delivery. This is related to the wide perception that disability is caused by witchcraft and spirit beings and can be transmitted if pregnant PWDs are allowed to come into contact with “innocent” others. This supports findings from several studies [1, 16, 19, 23, 26] and also confirms an earlier study in Ghana [16] which found that these beliefs existed and are perpetuated by the ubiquitous cultural and religious beliefs that disability is spiritually transmitted. It was also revealed that as over 60% of respondents have negative attitudes towards pregnant PWDs. Thompson et al. and Staniland have reported that almost all PWDs in their study reported to have been denied the right to exercise their SRH rights [24, 27]. Esmail et al. similarly in their qualitative study found out that PWDs are largely faced with negative public attitudes towards their sexuality and proposed that the rationale behind these negative public attitudes may be attributed to misconceptions, prejudices, and myths [15]. Researchers have reported how negative perceptions about the sexuality and marriage of PWDs have been one of the major perpetuators to negative perceptions and attitudes about women with disabilities and their pregnancies [10, 21].

It was especially of interest that while majority of respondents felt that PWDs should be able to have sexual relations, ironically, less than half of the same respondents felt comfortable with a close friend or relative having sexual relations with a PWD. A similar dissonance was observed with respect to marital relations.

While many women with disabilities desired to be pregnant and enjoy motherhood, society perceives that women with disabilities cannot have a positive motherhood experience. This was evidenced by a significant finding that came up in our study that society perceives women living with disabilities as incapable of having a safe motherhood experience.

There was evidence even where women with disabilities were not considered asexual; it was clear that sexual relationships do not extend into marriage. This is related to the mind-set that society has towards PWDs in general. For example, the public assumption is that people with disabilities are asexual or are simply seen as people who are incapable of marriage and child birth. We found out that even where women with disabilities were not considered asexual, it was clear that sexual relationships do not extend into marriage

About 60% of respondents would be uncomfortable to have a woman with a disability as a sister-in-law. This proved especially interesting as 94.5% of respondents initially indicated that they felt PWDs should be able to marry people without disabilities but were far less willing (39.2%) to accept a marriage between their close friend or relative and a PWD. Again, this relates to the perception that female PWDs in Ghana are often regarded as unproductive and incapable of contributing in a positive way to marriage and child birth.

Instead of being viewed as partners and family assets, they are rather seen as constituting either a physical, sociocultural or economic burden on the family. Their husbands are seen in the light of a burdened caregiver. Due to this, they are unlikely to be considered by society as best fit for marriage and onward transitioning to pregnancy and childbirth.

This phenomenon has been widely reported by other studies investigating attitudes of health personnel towards PWDs [10, 27, 28] where providers mistreated PWDs due to their belief that they were not entitled to sexual activity and questioned the capability of disabled women to have a positive motherhood experience [25]. It is obvious that some healthcare providers often times appeared ill-prepared to address the maternity needs of women with disabilities [29, 30]. This was evidenced in our study as PWDs reported some negative attitudes of some SRH professionals either during a personal experience during pregnancy or by witnessing a fellow PWDs go through unpleasant experiences from healthcare providers. Healthcare providers were reported to ignore pregnant PWDs and give priority to others, in anticipation of communication problems. This was especially noted among pregnant PWDs with speech and hearing impairments [10]. For expectant women with disabilities, these negative attitudes have been reported across several instances to have resulted in life-threatening situations for both mother and baby [21].

It was revealed that educational level of respondents had a significant association with negative attitudes and perceptions towards pregnant PWDs. Respondents who had attained at least secondary level of education were approximately twice more likely to have a negative attitude and perceptions towards pregnant women with disabilities as compared to those with at most primary level of education although previous studies reported contrary findings. Thompson et al. and Staniland in their studies in Britain both reported education having a positive correlation with positive attitudes towards PWD's as compared to the uneducated [23, 26]. Similarly, a study in Canada reported that current negative societal attitudes and perceptions about the sexual and reproductive rights of women living with disabilities are largely driven by lack of education and knowledge [15]. One plausible explanation for our findings may be that higher educated people generally perceive themselves of a higher social class and may not want to associate themselves with PWDs who are generally perceived as socially deprived, cognitively impaired and handicapped. Additionally, educated people may more logically consider the demands of pregnancy and may disapprove upon the pregnancy of PWDs as they may seem unfit to take care of themselves and their unborn child. For instance this study found in accordance with other studies that healthcare providers who are educated on pregnancy and its associated complications are more likely to abruptly classify a pregnant disabled woman as high risk without critical physical examination. This complements a study conducted by Ganle et al. in Ghana which also recorded that health care providers felt ill-prepared and uncomfortable to address the maternal health needs of pregnant PWDS in anticipation of pregnancy and child birth related complications [10].

The study found out that individuals with personal contacts or exposure to persons with disabilities were more likely than those who have no contact to have a negative attitude towards pregnant PWDs. The contrary is however reported in studies by Thompson et al., and Staniland where individuals (family members, neighbours or coworkers or friends) with consistent and recent exposures to PWDs tend to portray more positive attitudes towards PWDs compared to individuals with little or no exposures[23, 26]. A plausible reason for our finding is that, in establishing personal contacts with PWDs, it is likely that they may gradually become frustrated and irritated with them due to the continuous assistance they require in performing otherwise basic tasks. This is evidenced by a study in china by Qiaolan et al. [24], where caregivers who have had prolonged exposures or contacts with PWDs had more negative attitudes towards disability. Families and friends including caregivers provide uncompensated support and care to PWDs along with physical support that includes the performance of daily tasks for PWDs and even more of these when the PWD is pregnant. Mastering these tasks and providing the needed physical and emotional support for PWDs and even so during pregnancy may be very challenging. Given this increased strain on close relatives in Ghana, this may particularly impact even more negative attitudes towards pregnant PWDs. Awareness about the existence of laws that protects the rights of PWDs has been reported to be largely low in Ghana [12]. This was evidenced in our study which discovered that awareness of disability laws and rights was associated with negative attitude and perceptions towards pregnant women with disabilities. Although the convention on the rights of PWDs was ratified over a decade ago, there has been very little awareness on the existence of these laws and its provision. It was revealed that only 3.5 % of the respondents knew about the existence of disability laws and rights. Community members who were not aware of the laws and rights of PWDs were more likely to have a negative attitude towards pregnant PWDs as opposed to those who were aware of its existence. Similarly, another Ghanaian study by Slikker found out that only about 30% of individuals without disabilities were aware of the existence of disability laws and rights [16]. The greatest challenge in Ghana is that even people living with disabilities and their close relatives are unaware of these laws [12]. This has catalyzed the already existing discriminations towards PWDs as individuals may be unaware of their actions that may infringe upon the rights of PWDs and PWDs on the other hand may not realize as and when they are denied their basic rights in society.

With regard to the relationship between perceived cause of disability and the resultant attitudes, respondents were three times more likely to have negative attitude and perceptions towards pregnant PWD if their cause of disabilities was perceived to be spiritual compared to the cause being medical. In consonance, Reynolds, in a study on disability cultures in Ghana, also identified spiritual powers as a commonly reported cause of disability [19]. We attribute this finding to the social perception that disability is caused by witchcraft, spirits, or an angry god [16]. It is believed that pregnant women with spiritually caused disabilities may spiritually transfer witchcraft and curses to their unborn child or that of another woman. To avert such occurrences, pregnancy among PWDs is discouraged within communities. In the event where a PWD is found pregnant, she is subjected to different forms of discriminations and negative attitudes [16]. Also, respondents were up to five (5) times more likely to have not had a negative attitude and perception towards pregnant PWDs if the cause was perceived to have been due to accidents. This may be attributable to the general show of sympathy towards accident victims. This, coupled with being pregnant, may stimulate even more sympathy. This is also buttressed by the reported feelings of awkwardness (84.4%) or sorrow (87.1%) towards pregnant PWDs whose disabilities were caused by accidents.

## 6. Limitations

This study was conducted in a typically rural district and findings may not be necessarily representative of urban areas.

## 7. Conclusion

This study sought to describe personal experiences, community attitudes, and perceptions of pregnant women living with disabilities and how certain factors influence these attitudes and perceptions. On the basis of the findings of this study, a number of conclusions were drawn.

The evidence suggests that a degree of prejudice and misconceptions exists towards pregnant women living with disabilities. Generally, there is a public perception that women living with disabilities cannot have a safe motherhood experience and are capable of transferring their disability to the fetus of an expectant woman without a disability. In light of this, community attitudes and perceptions towards pregnant women with disabilities were largely negative which was found to have been driven by several factors. We discovered that perceived cause of disability (accidents and spiritual beliefs), high educational level, and the awareness of laws and rights about disability are the most significant factors that influence negative attitudes and perceptions of community members towards pregnant PWDs.

There is the need for government institutions such as the Ministry of Gender, Children, and Social Protection, disability help groups, and other stakeholders to raise public awareness about the SRH rights of women living with disabilities to combat disability stereotypes throughout society. In addition, government institutions and nongovernmental organizations need to promote interventions at local and national levels aimed at promoting sexual and reproductive health and rights and specifically target women living with disabilities. It is expedient for healthcare institutions to develop information accessible materials to PWDs.

## Figures and Tables

**Table 1:** Sociodemographic characteristics of respondents.

**Characteristics **	**Frequency**	**Percent (**%**)**
**Sex of respondents**		
Male	179	44.8
Female	221	55.2
**Age of respondents**		
18-25	117	30.1
26-30	123	31.6
31-35	55	14.1
36-40	50	12.9
>40yrs	44	11.3
**Marital status**		
Married	110	26.7
Widowed	7	1.8
Divorced	7	1.8
Cohabiting	169	42.6
Single	104	26.2
**Educational status**		
None	55	13.9
Primary	67	16.9
JHS	177	44.7
SHS	93	23.5
Tertiary	3	0.8
Others	1	0.3
**Employment status**		
Employed	298	74.5
Unemployed	102	25.5
**Ethnicity**		
Akan	6	1.5
Ewe	385	97.2
Ga	1	0.3
Others	4	1.0

**Table 2:** Participants knowledge about disability.

**Characteristics**	**Frequency**	**Percent (**%**)**
**Personal contact with a woman with disability**		
Yes	51	12.8
No	346	87.2
**Awareness of the Disability law or rights**		
Yes	14	3.5
No	383	96.5
**Perceived causes of disability (multiple responses) **		
Medical conditions	184	46.0
Accidents	343	85.8
Medical errors	58	14.5
Spiritual	355	89.4

**Table 3:** Distribution of how respondents felt when they met pregnant women with disabilities.

**Variable **	**Often**	**Sometimes**	**Rarely**	**Never**
Awkward/uncomfortable	69.9	14.5	6.3	9.3
Afraid of the person	19.5	26.5	15.3	38.8
Sorry for the person	60.3	26.8	8.8	4.3
Indifferent towards the person	10.8	28.0	18.3	43.0
Admiration for the person	4.8	12.8	20.8	61.8

**Table 4:** Respondents' perceptions about sexuality and pregnancy of female PWDs.

**Statements **	**Agree (**%**)**	**Disagree (**%**)**
(i) Women living with disabilities are treated fairly in Ghana	34.8	65.2
(ii) Pregnant Women living with disabilities should live in special institutions	95.7	4.3
(iii) Women living with disabilities can give birth to normal babies	100	-
(iv) Women living with disabilities can have children	99.5	0.5
(v) Women with disabilities are well integrated into maternal health care services	10.3	89.7
(vi) Women living with disabilities can have sexual feelings or desires	29.5	70.5
(vii) Women living with disabilities are asexual	60.8	39.2
(viii) People with disabilities are discriminated in Ghana	65.5	34.5
(ix) Women living with disabilities face double discrimination based on their sexuality or during pregnancy	80.5	19.5
(x)Pregnant women with disabilities should be not be kept away from other pregnant women in society	16.3	83.8
(xi) Pregnant women with disabilities are not capable of going through normal labor and childbirth	72.8	27.3
(xii) It is wrong for a woman with a disability to have children	14	86
(xiii) A woman with disability can marry a man without disability	94.5	5.5
(xiv) I will be comfortable if my close relative or friend has a sexual relationship with a woman living with disability	43.8	56.3
(xv) A woman with disability can only marry a man with disability	5.3	94.8
(xvi) A woman with disability can only have sexual relationship with a man with disability	5.3	94.8
(xvii) I would be comfortable if a woman with a disability was my sister in law	39.2	60.8
(xviii) Women living with disabilities should be kept apart from Society	13.5	86.5
(xix) Pregnant non - disabled women should avoid pregnant disabled women with disabilities because they can transmit the disability to their unborn child	56.3	43.8

**Table 5:** Factors influencing attitudes towards pregnancy in disability.

Variable	Community Attitudes towards Pregnant Women with Disabilities		X2
	Negative attitude	Positive attitude	*p-value*
**Sex **			
Male	127(70.9)	52(29.1)	0.074
Female	138(62.4)	83(37.6)	
**Age**			
18-25	67(57.3)	50(42.7)	0.115
26-30	81(65.9)	42(34.1)	
31-35	42(76.4)	13(23.6)	
36-40	32(64.0)	18(36.0)	
>40	32(72.7)	12(27.3)	
**Marital status**			
Married	73(66.4)	37(33.6)	0.591
Widowed	4(57.1)	3(42.9)	
Separated	5(71.4)	2(28.6)	
Cohabiting	118(69.8)	51(30.2)	
Single	63(60.6)	41(39.4)	
**Educational status**			
Primary and below	82(67.2)	40(32.8)	0.001*∗∗*
JHS	130(73.4)	47(26.6)	
SHS and above	49(50.5)	48(49.5)	
**Ethnicity**			
Akan	2(33.3)	4(66.7)	0.270
Ewe	257(66.8)	128(33.2)	
Ga	1(100.0)	0 (0.0)	
Other	2(50.0)	2 (50.0)	
**Employment status**			
Employed	204(68.5)	94(31.5)	0.111
Unemployed	61(59.8)	4(40.2)	
**Causes of disability **			
*Medical conditions*			
No	122(55.2)	99(44.8)	0.000*∗∗*
Yes	143(79.9)	36(20.1)	
*Accident *			
No	49(89.1)	6(10.9)	0.000*∗∗∗*
Yes	214(62.4)	129(37.6)	
*Spiritual causes *			
No	39(84.8)	7(15.2)	0.005*∗∗*
Yes	226(75.1)	128(24.9)	
*Medical errors*			
No	227(66.2)	116(33.8)	0.943
Yes	38(66.7)	19(33.3)	
**Awareness of disability laws and rights**			
No	259(67.3)	126(32.7)	0.057
Yes	6(42.9)	8(57.1)	
**Personal contact with a person with disability**			
No	224(64.2)	125(35.8)	0.022*∗*
Yes	41(80.4)	10(19.6)	

*∗∗∗p*<0.001;  *∗∗p*<0.01;  *∗p*<0.05.

**Table 6:** Logistic Regression of factors influencing attitudes towards pregnant women with disabilities.

Variable	Crude *OR* (95% CI)	*P*-value	Adjusted *OR* (95% CI)	*P*-value
**Educational status** (*ref*: Primary and below)				
JHS	0.74(0.45-1.23)	0.244	0.67(0.38-1.15)	0.145
SHS and above	2.00(1.16-3.48)	0.013*∗*	1.69(0.93-3.08)	0.084
**Sex of respondent** (*ref*: Male)				
Female	1.47(0.96-2.24)	0.074	1.37(0.85-2.21)	0.197
**Causes of disability**				
*Medical condition*(*ref*: No)				
Yes	0.31(0.20-0.49)	0.000*∗∗∗*	0.34(0.20-0.56)	0.000*∗∗∗*
*Accidents* (*ref:* No)				
Yes	4.92(2.05-11.81)	0.000*∗∗∗*	5.68(2.22-14.55)	0.000*∗∗∗*
*Spiritual cause*(*ref*: No)				
Yes	3.16(1.37-7.26)	0.007*∗∗*	2.05(0.85-4.98)	0.011*∗*
**Personal contact with a woman with disability **(*ref*: Yes)				
No	0.44(0.21-0.90)	0.025*∗*	0.68(0.31-1.49)	0.333
**Aware of disability laws and rights** (*ref*: Yes)				
No	2.74(0.93-8.07)	0.067	4.21(1.18-15.03)	0.027*∗*

*∗∗∗p*<0.001;  *∗∗p*<0.01;**  ***∗p*<0.05;  *OR *= odds ratio;  *CI *= confidence interval.

## Data Availability

Deidentified transcripts remain at School of Public Health, University of Ghana. The datasets and transcripts are available from the corresponding author upon reasonable request.

## Acronyms

AOR: Adjusted Odds Ratio
CI: Confidence interval
CRPD: Convention on the Rights of Persons with Disabilities
GHS-ERC: Ghana Health Service Ethical Review Committee
GPHC: Ghana Population and Housing Census
GSS: Ghana Statistical Service
IDIs: In-depth interviews
JHS: Junior High School
PWDs: People with disabilities
SHS: Senior High School
SRH: Sexual Reproductive Health
UNDP: United Nations Development Program
WHO: World Health Organization.
